# PD168 Developing And Implementing A Value Framework For Rapid Health Technology Assessments: Enhancing Evidence-Informed Coverage In Resource-Constrained Settings

**DOI:** 10.1017/S0266462324003945

**Published:** 2025-01-07

**Authors:** Andrea Alcaraz, Fernando Argento, Verónica Alfie, Sebastian Garcia Marti, Ariel Bardach, Agustín Ciapponi, Federico Augustovski, Andres Pichon-Riviere

## Abstract

**Introduction:**

Value frameworks play a crucial role in bridging the gap between evidence and decision-making in health care, particularly in settings with limited budgets. In this study, we present the results of an implemented value framework (VF) to provide coverage recommendations in rapid health technology assessment (HTA) reports.

**Methods:**

A search was performed to identify existing HTA frameworks. Relevant criteria and methods of assessment were then selected. A color-coded system was applied to categorize each criterion. A Delphi panel was conducted to determine the overall recommendations and to weigh the criteria and correlate them with the recommendations. To assess the performance, we reviewed the results of rapid HTA reports from the last five years.

**Results:**

The value framework had three domains. We adapted widely used methodologies for the quality of evidence and net benefit domains. The economic impact domain was the most complex to assess, so a customized method was developed. Analysis of 265 HTAs revealed the distribution of recommendations across various criteria and technology types. Most recommendations were for drugs (40.5%) or for therapeutic procedures (36%). Among the final recommendations, 0.8 percent were favorable, 19.7 percent were uncertain, and 44 percent were unfavorable.

**Conclusions:**

The VF demonstrated its versatility and practicality in meeting the needs of rapid HTA requesters and facilitating evidence-informed decision-making. The VF serves as a valuable tool for conducting adaptive rapid HTAs and supports decision-making processes in Argentina and similar contexts.

